# Alcoholic cardiomyopathy: an update

**DOI:** 10.1093/eurheartj/ehae362

**Published:** 2024-06-07

**Authors:** Fernando Domínguez, Eric Adler, Pablo García-Pavía

**Affiliations:** Department of Cardiology, Heart Failure and Inherited Cardiac Diseases Unit, Hospital Universitario Puerta de Hierro Majadahonda, IDIPHISA, CIBERCV, Manuel de Falla, 2, Majadahonda, Madrid 28222, Spain; Centro Nacional de Investigaciones Cardiovasculares Carlos III, Calle de Melchor Fernández Almagro, 3, Madrid, Spain; Section Head of Heart Failure, University of California, San Diego, CA, USA; Department of Cardiology, Heart Failure and Inherited Cardiac Diseases Unit, Hospital Universitario Puerta de Hierro Majadahonda, IDIPHISA, CIBERCV, Manuel de Falla, 2, Majadahonda, Madrid 28222, Spain; Centro Nacional de Investigaciones Cardiovasculares Carlos III, Calle de Melchor Fernández Almagro, 3, Madrid, Spain

**Keywords:** Alcoholic cardiomyopathy, Alcohol, Pathogenesis, Treatment

## Abstract

Alcohol-induced cardiomyopathy (AC) is an acquired form of dilated cardiomyopathy (DCM) caused by prolonged and heavy alcohol intake in the absence of other causes. The amount of alcohol required to produce AC is generally considered as >80 g/day over 5 years, but there is still some controversy regarding this definition. This review on AC focuses on pathogenesis, which involves different mechanisms. Firstly, the direct toxic effect of ethanol promotes oxidative stress in the myocardium and activation of the renin–angiotensin system. Moreover, acetaldehyde, the best-studied metabolite of alcohol, can contribute to myocardial damage impairing actin–myosin interaction and producing mitochondrial dysfunction. Genetic factors are also involved in the pathogenesis of AC, with DCM-causing genetic variants in patients with AC, especially titin-truncating variants. These findings support a double-hit hypothesis in AC, combining genetics and environmental factors. The synergistic effect of alcohol with concomitant conditions such as hypertension or liver cirrhosis can be another contributing factor leading to AC. There are no specific cardiac signs and symptoms in AC as compared with other forms of DCM. However, natural history of AC differs from DCM and relies directly on alcohol withdrawal, as left ventricular ejection fraction recovery in abstainers is associated with an excellent prognosis. Thus, abstinence from alcohol is the most crucial step in treating AC, and specific therapies are available for this purpose. Otherwise, AC should be treated according to current guidelines of heart failure with reduced ejection fraction. Targeted therapies based on AC pathogenesis are currently being developed and could potentially improve AC treatment in the future.

## Graphical Abstract

**Graphical Abstract:**
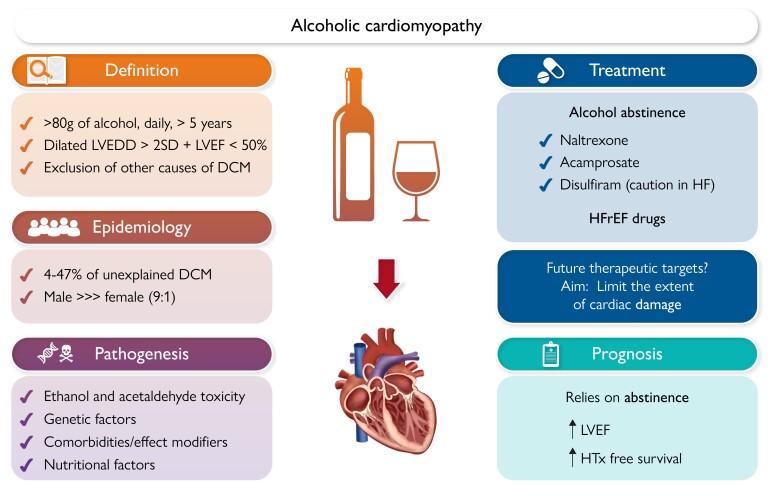
Clinical overview, pathogenesis, treatment and prognosis of alcoholic cardiomyopathy. DCM, dilated cardiomyopathy; HF, heart failure; HFrEF, heart failure with reduced ejection fraction; HTx, heart transplant; LVEDD, left ventricular end-diastolic diameter; LVEF, left ventricular ejection fraction; SD, standard deviation.

## Introduction

According to the World Health Organization's Global Status Report on Alcohol and Health, around 56% of the global population aged 15 years or older had consumed alcohol in 2018.^1^ However, this percentage can vary significantly by country and demographic group. Alcohol use can lead to a range of health and social issues, including cardiovascular diseases. One of the potential consequences is alcohol-induced cardiomyopathy (AC), an acquired form of dilated cardiomyopathy (DCM), caused by long-term heavy alcohol consumption (classically defined as >80 g daily over a period of 5 years or more^2–8^), in the absence of other causes of DCM. This condition is also known as alcoholic cardiomyopathy, but the term ‘alcohol-induced cardiomyopathy’ will be used throughout this review as it emphasizes the causal relationship between alcohol consumption and the development of cardiomyopathy.

There is a different susceptibility to alcohol in different populations. More specifically, the flushing reaction to alcohol in Asian subjects is a consequence of aldehyde dehydrogenase 2 (ALDH2) deficiency, which is responsible for a acetaldehyde accumulation.^9^

This review will provide an updated view of this condition, including its epidemiology, pathogenesis, diagnosis, and treatment (*Graphical Abstract*).

Definition of AC is as follows:

1. Heavy alcohol consumption (>80 g/day) for at least 5 years.
2. Left ventricular end-diastolic diameter >2 SD above normal and left ventricular ejection fraction (LVEF) < 50%.^10^
3. Exclusion of other causes of DCM, including hypertensive, valvular, and ischaemic heart disease.

A standard drink contains 10 g of alcohol, equivalent to 100 mL of wine, 300 mL of beer, or 40 mL of spirits.

Furthermore, alcohol consumption has also been classified in the literature by ranges of consumption as mild, moderate, and heavy drinking.^11^ In this regard, these categories have the following consumption thresholds that also differ according to sex.

- Mild drinking: consumption of <20 g/day in men and <10 g/day in women.
- Moderate drinking: consumption of 20–60 g/day in men, 10–40 g/day in women
- Heavy drinking: consumption >60 g/day in men and >40 g/day in women.

### Epidemiology

The prevalence of AC among patients with otherwise unexplained DCM has been reported to range from 3.8% to 47%,^12^ according to the very different characteristics of the studied cohorts. The lowest prevalence was obtained from a series of patients admitted for heart failure that included hypertensive and ischaemic heart disease as causes of left ventricular systolic dysfunction. This added to the fact that only patients with a consumption of >200 g/day of alcohol over 6 months were included, which may explain the low prevalence reported.^12^

On the other hand, studies have found that the prevalence of idiopathic DCM (iDCM) is between 23% and 47% in individuals who consume 80 g/day of alcohol for 5 or more years.^5–7,13^ A similar prevalence of 40% was observed in a study that lowered the consumption threshold to 40 g/day.^4^ Furthermore, the same study found that 40% of patients with DCM consumed more alcohol than the recommended weekly intake, compared with 24% of the non-DCM control group (*P* < .01).

The largest study to date linking alcohol consumption and cardiac remodelling was conducted in South Korea and included almost 50 000 participants who underwent echocardiographic evaluation and were classified into different groups according to daily alcohol consumption: lifetime never drinker, occasional (<1 g/day), light (1–15 g/day), moderate (15–30 g/day), heavy (30–60 g/day), and very heavy (>60 g/day) drinker. Very heavy alcohol drinkers’ group had significantly increased ventricular wall thicknesses and impaired left ventricular diastolic function compared with non-drinkers. Regarding LVEF, all groups presented normal mean values. However, heavy drinkers presented a significantly lower LVEF compared with non-drinkers (66.9 ± 5.7% vs. 67.7 ± 5.5, *P* for trend < .001).^14^ It is important to highlight the fact that within this Asian population, there is a very high prevalence of ALDH deficiency, which increases the levels of alcohol cardiotoxic metabolites. Therefore, these results may not apply to other populations with different ethnic composition.

It is interesting to note that there is a significant gender imbalance in the incidence of AC, with men being more frequently affected than women. A study revealed that the ratio of men admitted to the hospital for AC compared with women was 9:1.^2^ The cause of this imbalance is not yet fully understood and is likely multifactorial, possibly related to social factors (such as under-reporting of heavy alcohol consumption in women), physiological factors (such as differences in hepatic ethanol metabolism and lower tolerance to alcohol in women), or even differences in the threshold of alcohol consumption used to define AC, which does not vary according to sex. Women may require a lower daily dose of ethanol and a shorter duration of alcoholism than men to develop AC.^15,16^  *Table 1* shows clinical data from the published cohorts regarding AC.

**Table 1:** Cohorts of patients with alcoholic-induced cardiomyopathy published to date

	*n* (AC group)	Age, mean (SD)	Male sex (%)	Mean daily intake (g)	Alcohol intake duration (years)	LVEF (%)	LVEDD (mm)	Atrial fibrillation (%)
Mathews *et al*.^17^	33	46	95%	>115	>6	35^a^	51	12
Dancy *el al*.^18^	33	46 ± 13	63.6%	-	-	35 ± 7^a^	49 ± 8	-
Kupari *et al*.^19^	78	39	100%	294 (90–690)	13	30 ± 0.5^a^	26 ± 0.3^1^	-
Teragaki *et al*.^20^	20	54	100%	147 ± 17	31 ± 2	-	64 ± 0.2	-
Fernandez-Solá *et al*.^15^	36	50 ± 8	72.2%	194 ± 56	29 ± 6	32 ± 12	38 ± 7^1^	22.2
Ballester *et al*.^21^	56	46 ± 11	96.4	123 ± 60	21 ± 9	28 ± 12	71 ± 10	-
Guillo *et al*.^22^	14	45 ± 9	100	228 ± 61	13 ± 4	22 ± 6	69 ± 5	21.4
Gavazzi *et al*.^7^	79	45 ± 10	100	177 ± 96	14 ± 11	28 ± 7	38 ± 6^1^	15
Nicolás *et al*.^23^	55	48 ± 7	100	208 ± 56	27 ± 7	39 ± 11	62 ± 9	20
Guzzo-Merello *et al*.^24^	94	50 ± 10	99	>80	>5	26 ± 9	68 ± 9	34
Ram *et al*.^2^	45 365 (admissions)	45–59	87	-	-	-	-	31.2
Amor-Salamanca *et al*.^25^	101	50 ± 10	99	>80	>5	26 ± 9	67 ± 10	37.6
Fang *et al*.^26^	299	51 ± 11	99	>80	>5	35 ± 11	68 ± 9	13.7
Dundung *et al*.^27^	290	53 ± 22	57	>80	>5	31 ± 11	60 ± 1	38
Manivannan *et al*.^28^	1237	59 ± 10	93	>80	>5	-	-	33

AC, alcohol-induced cardiomyopathy; LVEF, left ventricular ejection fraction; LVEDD, left ventricular end-diastolic diameter.

^a^Fractional shortening. 1: LVEDD index (mm/m^2^).

#### Alcoholic consumption and heart failure

Some years ago, it was believed that the relationship between alcohol consumption and heart failure followed a J-shaped pattern.^29^ Several studies observed a beneficial effect with low-to-moderate alcohol use, with a lower risk of developing heart failure in those taking 7–14 drinks per week.^30–33^

However, more recent studies have discarded the beneficial effect of lower levels of consumption based on methodological issues that may have biased the results and conclusions of older studies.^34^ In this regard, it has been observed that light to moderate drinking is associated with a healthier lifestyle, higher socio-economic status and a more active family and social life, as opposed to a heavier intake of alcohol.^35^ On the other hand, a combined analysis of 83 studies including almost 600 000 subjects found that an alcohol consumption of >100 g/week is associated with an increased risk of heart failure and lower life expectancy.^36^

There is some controversy regarding the amount of alcohol required to produce AC, but it is generally considered as >80 g/day over 5 years.^2–8^ In 1974, Koide and Ozeki were the first to report a relationship between alcohol consumption and cardiomegaly in chest X-rays. Their study showed that one-third of subjects who consumed more than 125 mL of alcohol per day (type not specified) exhibited cardiomegaly, compared with only 5.7% in mild consumers.^37^ Later, Askanas *et al*. reported an increase in myocardial mass in subjects who consumed more than 120 g of alcohol per day as compared with non-drinkers. Surprisingly, there were no differences found between 5- and 14-year drinkers and those who had consumed alcohol for more than 14 years.^38^

A systematic review published in 2017 aimed to establish an alcohol dose threshold to cause AC.^39^ Unfortunately, the lack of sufficient data did not permit the computation of pooled estimates through meta-analyses. In any case, heavy drinking (≥80 g/day) along with bigger lifetime alcohol exposures was clearly associated with a higher risk of cardiomyopathy.^39^

### Pathogenesis of alcohol-induced cardiomyopathy

**Figure 1:**
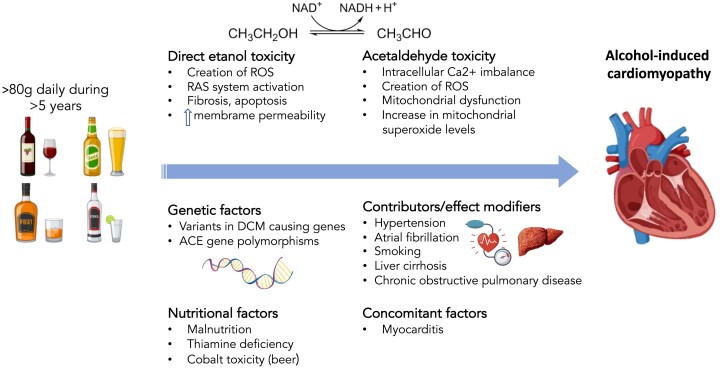
Pathogenic mechanisms of alcohol-induced cardiomyopathy. ACE, angiotensin-converting enzyme; DCM, dilated cardiomyopathy; RAS, renin–angiotensin system; ROS, reactive oxygen species

The pathophysiology of AC involves a combination of direct toxic effects of alcohol on the myocardium, oxidative stress, mitochondrial dysfunction, and genetic susceptibility. Ethanol and its metabolites can disrupt cellular processes, impair protein synthesis, and cause oxidative stress, leading to cellular injury and dysfunction within the heart muscle.^40^ Moreover, the pathogenesis of AC also involves genetically related factors and indirect mechanisms such as nutritional deficits (*Figure 1*).

#### Direct toxic effect of ethanol

Ethanol can promote oxidative stress in the myocardium contributing to the creation of reactive oxygen species (ROS) or activating additional systems, such as renin–angiotensin system (RAS).^41^

The direct toxic effect of ethanol has been studied in both acute ‘binge’ drinking and chronic intake. A mouse model on a diet consisting of 5% alcohol and a single episode of 5 g/kg body weight of binge drinking exhibited mitochondrial dysfunction, contractile impairment, and decreased total peripheral resistance after 10 days.^42^ In humans, binge drinking causes reversible changes that can be detected by cardiac magnetic resonance imaging as soon as 24 h after excessive alcohol consumption, when acute drinkers present a significant increase in median myocardial T2-signal intensity as compared with non-drinkers.^43^ However, LVEF or ventricular volumes do not seem to be affected by binge drinking. In any case, repeated episodes of acute heavy drinking can eventually cause hypertension and are a risk factor for myocardial infarction and stroke.^44^

Chronic alcohol intake is directly toxic to cardiac myocytes, causing cell death, fibrosis, and impaired contractility in both animal models and humans.^11^ In mice treated with alcohol for 90 days, it has been observed that fibrogenesis might be explained by an excessive T helper 2-cell response to ethanol.^45^ Moreover, *Figure 1* depicts the effect of ethanol activating NOX2 and the subsequent oxidation of Ca/calmodulin-dependent kinase II (CaMKII). This results in sarcomeric reticulum Ca^2+^ leak, which is one of the causes of arrhythmogenic and negative inotropic effects.^46^

Ethanol consumption also activates the RAS, causing progressive cardiac dysfunction.^41^ In this context, the blockage of angiotensin II type 1 receptor with irbesartan has been shown to prevent the development of AC in a dog model.^47^ Additionally, the activation of mitochondrial ALDH2 has been found to have a protective effect by inhibiting the local cardiac RAS through the p38 MAPK/CREB pathway in an AC mouse model.^48^

#### Acetaldehyde damage

The best-studied toxic metabolite of alcohol is acetaldehyde, produced by alcohol dehydrogenase in the liver.^49^ Acetaldehyde cannot be synthesized directly by the cardiomyocyte or in muscle cells. Thus, its presence in the myocardium can only be explained from its diffusion from the bloodstream, if it has been possible to avoid the action of acetaldehyde dehydrogenase that breaks it down into acetate in the liver.^49^

Due to its significant toxicity, studies have avoided its direct instillation, as it produces indiscriminate cell damage even at low doses. Alternatively, studies have analysed its effect by combining ethanol with cyanamide. This substance is a potent inhibitor of the enzyme acetaldehyde dehydrogenase, so it increases the presence of acetaldehyde, and it promotes its effects.^48,50^ The harmful effects of this substance have been found to be exerted at various levels, in both animal and human models. It reduces the supply of calcium from the sarcoplasmic reticulum to the cytoplasm, decreasing protein synthesis and impairing the cellular excitation–contraction link and the interaction between actin and myosin.^51,52^ Moreover, acetaldehyde produces mitochondrial dysfunction, with the generation of ROS contributing to oxidative stress and causing changes in the mitochondrial oxidative phosphorylation system^53^ (*Figure 1*).

In fact, Brandt *et al*.^54^ observed that in ALDH2-deficient mice, the most important increase in mitochondrial superoxide levels (which is the major species of ROS) is due to acetaldehyde, not ethanol. By inhibiting NOX2 (the most important superoxide-producing enzyme) with apocynin, they observed a decrease in ethanol- and acetaldehyde-induced superoxide levels. Therefore, NOX2 could be a target for future therapies in AC.

#### Genetic factors

In the last years, genetic factors have acquired an important role in the pathogenesis of AC. Back in 2002, an angiotensin-converting enzyme (ACE) gene polymorphism was reported to be associated with vulnerability to AC. The authors compared the ACE genotypes of AC patients with a group of alcohol abusers without signs of cardiomyopathy and found a polymorphism in 57% of AC subjects vs. 7% in the non-AC group.^55^ Later, Lemieux *et al*.^56^ described a cohort of 237 HER2-positive breast cancer patients treated with trastuzumab, in which the HER2 Ile/Val polymorphism and alcohol intake (<10 drinks/80 g/week) were risk factors for cardiotoxicity.

More recently, our group showed that the prevalence of rare variants in DCM-causing genes was similar in non-alcoholic DCM vs. AC, with a predominance of titin-truncating variants (TTNtv).^57^ Alcohol-induced cardiomyopathy patients with a TTNtv had an 8.7% absolute reduction in LVEF compared with those with AC but without TTNtv paving the way to support the double-hit hypothesis of DCM arousing from the combination of genetics with environmental factors (*Figure 1*). This theory has gained support lately with the description of similar interaction between rare genetic variants and other factors like chemotherapy and myocarditis.^58,59^

#### Comorbidities

The synergistic effect of alcohol with concomitant conditions that do not cause DCM exclusively by themselves can be another contributing factor leading to AC development. Alcohol-induced cardiomyopathy patients exhibit a high prevalence of hypertension, smoking, atrial fibrillation, and chronic obstructive pulmonary disease.^60^

More specifically, atrial fibrillation with rapid ventricular response is a cause of arrhythmia-induced cardiomyopathy,^61^ which can potentially worsen LVEF in AC patients, on top of the direct toxic effect of ethanol, acetaldehyde damage, or the aforementioned genetic factors.

Moreover, it has been suggested that extracardiac comorbidities such as liver cirrhosis or protein-calorie malnutrition increase the risk of developing AC.^62^ As for liver cirrhosis, it has been proposed that the release of inflammatory cytokines such as tumour necrosis factor-α, interleukin-1β, and interleukin-6 might contribute to cardiac damage as these cytokines are increased in cardiac tissue of cirrhotic animal models.^63^ This syndrome, known as cirrhotic cardiomyopathy, is sometimes misdiagnosed as some symptoms are attributed to advanced cirrhosis^64^ Moreover, liver cirrhosis can cause portopulmonary hypertension, leading to pulmonary artery vasoconstrictions and haemodynamic failure with right-sided heart failure.^65^

#### Nutritional factors

Chronic alcohol consumption often leads to malnutrition and deficiencies in essential nutrients like thiamine (vitamin B1), folate, or magnesium.^66^ These deficiencies were thought to be instrumental in AC pathogenesis in the past, but both animal and human models controlled by dietary and anthropometric variables have verified that the cardiac-depressant effect of alcohol is independent of these nutritional factors.^18,67^ For instance, beriberi (severe vitamin B1 deficiency) causes heart failure with increased cardiac output, as opposed to AC, and clinical manifestations usually correspond to fluid retention in the context of right-sided heart failure.^68^

Another nutritional factor classically involved in the pathophysiology of AC was cobalt excess. The ‘Quebec beer drinkers' cardiomyopathy’ was related to cobalt supplementation to beer that was made in the past. It was described as a form of DCM with severe pericardial effusion, low cardiac output, and purplish skin coloration. Although initially attributed to alcohol, cobalt turned out to be entirely responsible of the disease as cobalt was found to compete at the cellular level with calcium and magnesium and appeared to alter both fatty acid metabolism and the pyruvate pathway.^69,70^ This syndrome disappeared completely when cobalt was removed from the brewing process.

### Clinical manifestations and diagnosis of alcohol-induced cardiomyopathy

There are no specific cardiac signs and symptoms in AC as compared with other forms of DCM. Onset symptoms can include acute shortness of breath, orthopnoea, and paroxysmal nocturnal dyspnoea, although some patients may have a sub-acute clinical presentation showing dyspnoea on exertion. Low cardiac output symptoms including fatigue and weakness may be present. In more chronic cases, signs of right-sided heart failure as oedema, jugular venous distention, and hepatomegaly start to develop. Jugular venous pressure can help with the differential diagnosis of cirrhosis in these patients (in the absence of tense ascites that can increase intrathoracic pressure).^8^

Supraventricular tachyarrhythmias are common in AC and patients can present with palpitations or dizziness, as well as cardiogenic syncope. Atrial fibrillation is the most common arrhythmia in AC, with a similar prevalence as compared with iDCM. Of note, in a modern series of patients with AC, Guzzo-Merello *et al*.^24^ reported 34% of atrial fibrillation within AC patients compared with 24% in non-alcohol-related DCM. Regarding ventricular arrhythmia, alcohol intake does not seem to worsen the prognosis on ventricular arrhythmias in patients with AC compared with those with iDCM.^71^

#### Blood tests

Along with signs of heart failure such as increased N-terminal pro-B-type natriuretic peptide, blood tests can provide hints suggesting chronic alcohol abuse. Blood test abnormalities that can raise the suspicion of AC in a patient with systolic dysfunction include elevated liver enzymes (alanine aminotransferase and aspartate aminotransferase), increased gamma-glutamyl transferase, macrocytosis (elevated mean corpuscular volume), anaemia (including folate and vitamin B12 deficiency), thrombocytopenia, hypertriglyceridaemia, hyperuricaemia, hypoalbuminaemia, or electrolyte imbalances (hypomagnesaemia, hypokalaemia, or hypophosphataemia).

#### Electrocardiogram (ECG)

ECG abnormalities are not specific in AC, but common findings include atrial fibrillation (14%–38% of patients),^58^ conduction abnormalities (left or right bundle branch block, haemiblocks, different degrees of atrioventricular blocks, ST/T wave changes, and QT interval prolongation.^60^ The latter has been linked to ionic disturbances such as hypokalaemia or hypomagnesaemia.^72^

#### Echocardiogram

**Figure 2:**
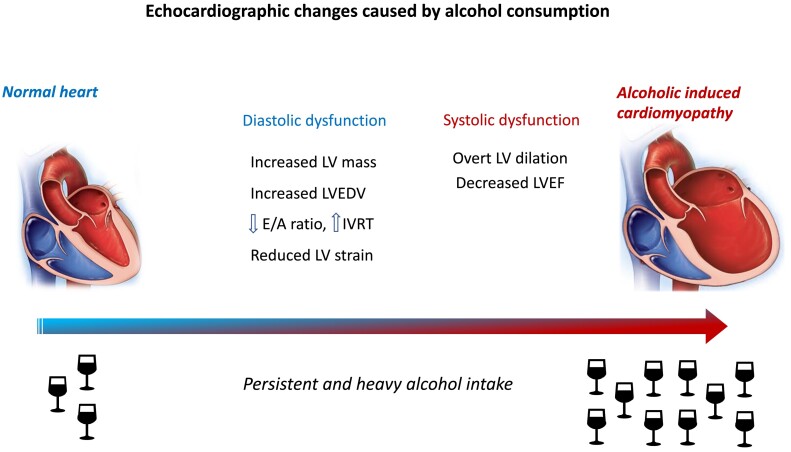
Echocardiographic consequences of heavy and persistent alcohol consumption. IVRT, isovolumic relaxation time; LVEDV, left ventricular end-diastolic volume; LVEF, left ventricular ejection fraction

Left ventricular dilation with impaired systolic function is key diagnostic echocardiographic features in AC, and there are no pathognomonic features to differentiate it from iDCM. Back in the 1980s, Urbano-Marquez *et al*.^73^ showed that the decrease in LVEF among alcoholics was clearly related to the accumulated alcohol intake throughout the year. However, even patients with alcohol use disorders and no cardiac symptoms present subclinical abnormalities. It has been described that almost 50% of these subjects present increased left ventricular mass with preserved LVEF.^38^ More recently, several studies have described increased left ventricular end-diastolic volume as an early cardiac abnormality related to alcohol abuse, as well as decreased *E*/*A* ratio and prolonged isovolumic relaxation time^74,75^ (*Figure 2*). Other studies with more sensible techniques to detect initial systolic dysfunction like speckle tracking analysis have reported subclinical decrease of systolic function in alcohol abusers.^76^ In this regard, subjects with >150 mg of daily ethanol intake present lower rotation and twist values compared with moderate and mild drinkers.^76^ Moreover, left ventricular global longitudinal strain and left ventricular global circumferential strain have been observed to be lower in alcohol abusers compared with controls.^77^

#### Cardiac magnetic resonance

As with echocardiography, there are no specific features of AC in cardiac magnetic resonance imaging. When compared with iDCM, a study has shown a lower LVEF and larger left ventricular end-diastolic volume in AC.^78^ Also, late gadolinium enhancement was predominantly mid-wall and septal in AC vs. lateral in iDCM group.^78^ More recently, Tayal *et al*. showed that patients with DCM assessed with cardiac magnetic resonance who had a history of moderate excess alcohol consumption (>168 g/week for men and >98 g/week for women) have increased biventricular and left atrial volumes, as well as worse left ventricular systolic function and hypertrophy than DCM patients without moderate excess alcohol consumption. However, these differences disappeared after adjusting for sex, as the group with at least moderate alcohol consumption was mainly made up of males. Moreover, there was no difference in mid-wall myocardial fibrosis either.^79^

#### Role of endomyocardial biopsy in alcohol-induced cardiomyopathy

Alcohol-induced cardiomyopathy does not represent an indication for endomyocardial biopsy according to the recent position statement by the Heart Failure Association of the European Society of Cardiology, Heart Failure Society of America, and Japanese Heart Failure Society.^80^ However, it can be considered in patients with DCM whose LVEF does not improve with alcohol abstinence and heart failure drugs, after discarding a genetic aetiology and ischaemic heart disease.

Previous reports indicate that ethanol can inhibit neutrophil chemotaxis and suppress cytokine production, which regulates inflammatory responses to infectious agents.^81^ In this regard, histologically confirmed myocarditis has been found in patients with suspected AC.^82^

### Natural history and prognosis of alcohol-induced cardiomyopathy

The prognosis of AC relies directly on alcohol withdrawal. Different studies have shown worse outcomes in patients who continue to drink as compared with other causes of DCM.^6,7^ In the early 2000s, Fauchier *et al*.^6^ compared a cohort of 50 patients with AC with 84 patients with iDCM and found that AC male subjects who did not stop drinking had an increased mortality compared with iDCM. Other series of patients with AC and iDCM support these results, with a significantly lower transplant-free survival in AC compared with iDCM.^7^

A more recent study by our group that included 94 patients with AC and 188 with iDCM found that AC was associated with a favourable outcome with a higher heart transplantation-free survival (15% in AC vs. 35% in iDCM during a median follow-up of 59 months).^24^ This study also showed a similar prognosis in AC abstainers and those who persisted with a moderate alcohol intake (<80 g/day). Among patients with AC, atrial fibrillation, QRS > 120 ms, and lack of beta-blocker therapy were independent predictors of death or heart transplantation.^24^ Moreover, in the long term, LVEF recovery is associated with an excellent prognosis in AC.^83^

Another study by our group focused on malignant ventricular arrhythmia in AC as compared with iDCM and showed that left bundle branch block and alcoholic aetiology were the only independent predictors of these arrhythmic events, which included sudden cardiac death (SCD), cardiopulmonary resuscitation, or appropriate implantable cardiac defibrillator (ICD) therapy.^84^

Regarding SCD in AC, a recent study specifically addressed this issue. Alcohol-induced cardiomyopathy was the cause of 4.9% of SCD (*n* = 290 out of 5869 victims), but only 22.1% of these subjects had been diagnosed of AC during life.^85^

### Treatment of alcohol-induced cardiomyopathy

Alcohol-induced cardiomyopathy treatment includes a combination of lifestyle modifications, pharmacological treatment, management of arrhythmia, and supportive care.

#### Lifestyle modifications

Abstinence from alcohol is the primary and most crucial step in treating AC.

Over the years, different observational studies have addressed this issue and proved that left ventricular function improves with abstinence, including periods from 10 weeks to 1.5 years.^83,86–89^ In this regard, Haissaguerre *et al*. retrospectively analysed the evolution of 126 individuals diagnosed with iDCM and 110 with AC (alcohol consumption of >80 g/day). After a mean follow-up of 38.8 ± 27 months, mortality was significantly less frequent in patients with AC who maintained abstinence than in the group without abstinence and in the group with iDCM (6.1% in those who maintained abstinence vs. 50.8% in those who did not abstain and 36.2% in those with iDCM). However, it is relevant that those patients who continued to drink alcohol moderately (<80 g/day) also presented a better prognosis.^13^

More recently, we reported that in a series of 101 patients with AC followed during a median of 82 months, none of those who persisted with heavy alcohol intake showed LVEF recovery. However, among the 42% of patients who significantly improved LVEF, there was no difference in recovery rate between abstainers and those who continued drinking but at moderate or low levels.^25^ In this same study, beta-blocker treatment, QRS < 120 ms, and absence of diuretics were independent predictors of LVEF recovery.

Although complete abstinence from alcohol is recommended to improve prognosis in AC, some studies have also observed an increase in LVEF in those patients who control their alcohol consumption to <80 g/day.^23,24^

Other lifestyle modifications recommended in AC patients include a low-sodium diet with a variety of fruits, vegetables and whole grains, fluid restriction, and regular exercise, an in other patients with DCM and heart failure.^90^

Unfortunately, it is well known that abstinence is difficult to achieve, and it is important to stress that alternative treatments are needed, including therapies to help with alcohol withdrawal, heart failure drugs, and other promising therapeutic approaches that focus on pathogenesis.

#### Pharmacological treatment for alcohol use disorder

Some drugs can help to achieve abstinence in alcohol use disorders, including ALDH inhibitor disulfiram, opioid antagonist naltrexone, and non-competitive N-methyl-D-aspartate-receptor blocker acamprosate.^91^ A randomized, multi-centre, open-label trial compared these three drugs and showed that abstinence days were significantly more frequent in subjects who took disulfiram.^92^ More recently, a Swedish nationwide study with more than 125 000 patients showed that naltrexone, as both monotherapy and combined with disulfiram or acamprosate, was more effective in reducing hospitalizations for alcohol-related causes.^93^ Naltrexone and acamprosate do not present cardiac-related contraindications.^94^ However, disulfiram can only be used with caution in mild AC cases without overt heart failure, as it is contraindicated in these patients. A potential disulfiram–alcohol reaction enhances palpitations, tachycardia, chest pain, or hypotension, especially dangerous in patients with heart failure.^95^

#### Pharmacological treatment for heart failure

Patients with AC should be treated according to current guidelines as any patient with heart failure with reduced ejection fraction.^96–98^ Pharmacological treatment includes beta-blockers, angiotensin receptor–neprilysin inhibitors/ACE inhibitors/angiotensin II receptor blocker, mineralocorticoid receptor antagonists, and sodium–glucose cotransporter-2 inhibitors. Additionally, diuretics are used to control fluid retention. The response to heart failure drugs can be variable among different patients with AC, and their effectiveness may be limited if alcohol consumption is maintained.^25^

No contemporary studies have specifically addressed the role of each of these drugs in AC. However, a study published in 2016 showed the benefit of carvedilol and trimetazidine added to conventional HF therapy that included ACE inhibitors, spironolactone, diuretics, and digoxin. After 12 weeks of therapy, patients receiving carvedilol and trimetazidine showed higher LVEF and longer walking distance in the 6-min test.^99^ Moreover, the absence of beta-blockers as a marker of worse prognosis in AC supports the important role of these drugs in AC.^24^

#### Management of arrhythmia

Alcohol consumption is related to an increased incidence of arrhythmia: both supraventricular (mostly triggered by binge drinking in younger patients) and ventricular tachycardia (in heavier drinkers).^100^ However, in AC patients, alcohol intake does not seem to worsen prognosis regarding ventricular arrhythmia compared with iDCM.^71^ In any case, abstinence lowers the risk of SCD and ventricular arrhythmia in AC.^71^

The indications for ICD or cardiac resynchronization therapy are the same as in non-ischaemic DCM,^96,97,101^ excluding those cases with an underlying high-risk genetic variant that prompts a lower threshold for ICD implantation (e.g. *LMNA*, *RBM20 FLNC*, and other high-risk genes).^102^ The same applies for the medical management of ventricular and supraventricular arrhythmia in these patients, which is outside the scope of this review.

#### Supportive care

Extracardiac effects of alcohol such as liver cirrhosis, malnutrition, vitamin deficiency, or electrolyte imbalance need to be specifically treated by the corresponding specialists in patients with AC. The result of controlling these comorbidities, as well as concomitant cardiovascular risk factors that are frequent in this population (smoking, hypertension, diabetes, and dyslipidaemia), results in an improved prognosis of these patients.^11^ As an example, an early intervention regarding excessive alcohol intake can have a positive effect on controlling hypertension.^103^

In cases of end-stage AC that do not improve with abstinence from alcohol or medical treatment, cardiac transplantation is the only remaining therapeutical option. Once complete abstinence from alcohol is achieved, heart transplantation has proven to be feasible in AC. In a series of 94 patients with AC, almost 30% underwent heart transplantation.^24^

#### Future therapeutic approaches: focus on pathogenesis

New therapeutic strategies for AC are being developed with the support of animal models. As the pathogenesis of AC is complex, specific treatments focus on different targets. These include damaging factors such as acetaldehyde or ROS, cardiac fibrosis, or apoptosis.

One of the strategies to limit the extent of cardiac damage is to decrease acetaldehyde levels. This can be achieved by activation of mitochondrial ALDH2 with Alda-1, as it accelerates acetaldehyde metabolism.^104^ Alda-1 has already been proven to reduce infarct size by 60% when administered to rats before an ischaemic event, inhibiting cytotoxic aldehydes.^105^ Moreover, transgenic overexpression of ALDH2 is protective against hypertrophy and contractile dysfunction in mice with chronic alcohol intake.^106^

Cardiac fibrosis related to AC has been targeted with soluble epoxide hydrolase inhibition in mice. Soluble epoxide hydrolase pharmacological inhibition blocked the activation of cardio fibroblasts induced by ethanol and contributed to the restoration of the autophagic flux suppressing mamalian target of rapamycin activation.^107^

**Figure 3:**
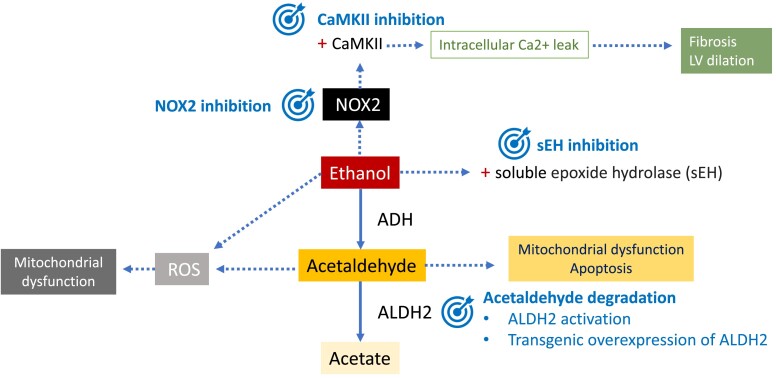
Potential therapeutic targets in alcohol-induced cardiomyopathy. Ethanol has direct toxic effects in the heart, as well as acetaldehyde, which is its most important metabolite. Ethanol is metabolized to acetaldehyde by alcohol dehydrogenase. Aldehyde dehydrogenase is responsible for the oxidation of acetaldehyde and its conversion into acetate. Ethanol produces activation of NOX2 and subsequent oxidation of Ca/calmodulin-dependent kinase II, inducing intracellular calcium (Ca^2+^) leak. Ca/calmodulin-dependent kinase II has been related to ethanol-induced cell death, with consequent fibrosis and dilation. Thus, calmodulin-dependent kinase II inhibition is an important therapeutic target. Ethanol also activates soluble epoxide hydrolases that turn epoxyeicosatrienoic acids into to less bioactive diols. Epoxyeicosatrienoic acids have cardioprotective properties, and soluble epoxide hydrolase inhibition can promote them. Both ethanol and acetaldehyde induce reactive oxygen species molecules and NOX2, which can be potentially targeted. Another therapeutic target in alcohol-induced cardiomyopathy is acetaldehyde degradation, by aldehyde dehydrogenase 2 activation or overexpression. AC, alcohol-induced cardiomyopathy; ADH, alcohol dehydrogenase; ALDH, aldehyde dehydrogenase; CaMKII, Ca/calmodulin-dependent kinase II; LV, left ventricle; ROS, reactive oxygen species; sEH, soluble epoxide hydrolase

Mitochondrial CaMKII has been related to ethanol-induced cell death, and its inhibition is shaping up as a potential therapeutic option (*Figure 3*).^108^

Murine models of myocardial CaMKII overexpression have shown ventricular dilation and hypertrophy, increased cell death, and fibrosis,^109^ whereas mice with genetic CaMKII inhibition are protected from left ventricular dysfunction and dilation after myocardial infarction.^109^ Recently, an orally available CaMKII inhibitor (RA306) has been used in mice with DCM carrying a pathogenic genetic variant in alpha-actin. RA306 significantly improved LVEF and cardiac output compared with placebo.^110^ Moreover, ethanol acutely activates CaMKII and leads to calcium leak from the sarcoplasmic reticulum favouring arrhythmia.^46^ In this context, CaMKII inhibitors could potentially be a therapeutic option for patients with AC. Another orally available CaMKII inhibitor, RA608, has shown a significant reduction in sarcoplasmic reticulum calcium leak in human atrial cardiomyocytes, together with reduced diastolic tension. In mice, a single oral dose reduced the inducibility of atrial and ventricular arrhythmia.^111^

Moreover, ranolazine prevents ethanol-induced atrial arrhythmias both *in vitro* and *in vivo* by blocking the late sodium current, which is activated by CaMKII.^112^ Its effect on preventing the decrease of LVEF in AC is currently unknown.

## Conclusions

Alcohol-induced cardiomyopathy remains a relevant health problem, for which the mainstay of treatment is alcohol abstinence. In recent years, basic and clinical research has shed light on its pathogenesis, which includes direct toxic effects of alcohol on the myocardium, oxidative stress, mitochondrial dysfunction, and genetic susceptibility.

Based on these valuable scientific contributions, new lines of research with targeted therapies are currently being developed.

## Data Availability

No data were generated or analysed for this manuscript.
